# Microplastic-Mediated Delivery of Di-butyl Phthalate Alters *C. elegans* Lifespan and Reproductive Fidelity

**DOI:** 10.3390/microplastics4040096

**Published:** 2025-12-01

**Authors:** Chiara Angelyn O. Maldonado, David M. Mares, Paola C. Garcia, Maria F. Gamez, Midori R. Flores, Alyssa D. Friudenberg, Ryan L. Peterson, Jennifer C. Harr

**Affiliations:** 1 Department of Biological Sciences, St. Mary’s University, San Antonio, TX 78228, USA; 2 Department of Chemistry and Biochemistry, Texas State University, San Marcos, TX 78666, USA

**Keywords:** microplastic pollution, microplastic, nanoplastic, ingestion, quantitative assessment, environmental exposure, human health, di-butyl phthalate, co-exposure, stress response, reproductive toxicity, polystyrene

## Abstract

Microplastics harbor chemical additives and absorb pollutants from the environment. Microplastics pose a human health threat and have been found in nearly all human tissues. The toxicological pathways and physiological effects of microplastic-mediated chemical exposure following ingestion remain unknown. Here we use *Caenorhabditis elegans* to investigate the effects of di-butyl phthalate and polystyrene microplastic mixtures on fertility and lifespan. Our studies demonstrate that 1 μm microplastics at 1 mg/L exposure levels result in decreased brood size, whereas 1000 times fewer microplastics (1 μg/L) did not affect the number of eggs laid. While there was no change in brood size at 1 μg/L microplastic exposure levels, there was an increase in embryonic lethality. Microplastics-mediated delivery of di-butyl phthalate to *C. elegans* significantly reduced brood size and increased embryonic lethality compared to exposure to microplastics alone. This reproductive toxicity is potentially due to a stress response via DAF-16, as observed with microplastics and di-butyl phthalate co-exposure. Furthermore, chronic exposure (from hatching onward) to microplastics shortened the lifespan of *C. elegans*, which was further reduced with di-butyl phthalate co-exposure. The exacerbated defects observed with co-exposure to phthalate-containing microplastics underscore the risks associated with microplastics releasing the additives and/or chemicals that they have absorbed from the environment.

## 1. Introduction

Plastic and chemical pollutants are ubiquitous in the environment, posing an emerging human health concern. Microplastic (MP) particles have been identified in nearly all human tissues, from blood [1] to the brain [2], and most recently in human reproductive organs [3,4]. MP exposure includes plastics from various environmental sources and exists in a wide range of chemical types, mixtures, and conditions. Primary MP particles are manufactured and used as ingredients in biomedical products and various health and beauty items (i.e., scrubs, make-up, cleansers, and toothpaste) [5], whereas secondary MPs result from the breakdown of larger plastic items. Human exposure to MPs occurs through direct use of MP-containing products, exposure to secondary MPs released into food, or environmental pollutants.

Both primary and secondary MPs undergo chemical and physical changes in their structure due to environmental exposure. Factors such as UV light, physical breakdown, and exposure to environmental pollutants can lead to alterations in the size, surface structure, and chemical composition of MPs. Despite efforts to reduce plastic use, it remains prevalent, with much already having entered the ecosystem [6]. Dating back to the 1950s, approximately 400 million tons of plastic waste accumulate each year [7]. MPs (MPs, plastic particles < 5 mm [8]) and nanoplastics (NPs, plastic particles ≤ 1 μm [8]) are detected in the air, water, and our food sources [9–13]. Meanwhile, MPs have been found in tissue exhibiting the ability to enter mammalian cells [14]. The smallest particles, NPs, invade endosomes, lysosomes, lymph and circulatory systems, and the lungs [15–17], leading to deleterious effects on the cellular level [16,17]. The direct health risks of human ingestion of MPs have not been quantified. Moreover, these risks amplify as plastics already contain additives, and they can absorb, accumulate, and transfer chemicals from the surrounding environment into organisms.

Plastic and resulting MP particles can be composed of homogenous or heterogeneous polymer mixtures, contain additives from the manufacturing process (plasticizers, by-products, and monomers), and may absorb chemical pollutants [18–20]. These additives and pollutants can later leach into the environment [21]. Persistent organic pollutants have been shown to be transferred from plastic particles to fish with adverse effects in environmentally relevant concentrations [22] and led to a transfer of chemicals from MPs into the guts of lung worms (*Arenicola marina*), and a reduction in biological functions [23]. In mice, it was demonstrated that phthalate esters were released from MPs leading to intestinal permeability and inflammation [24]. A study using *Caenorhabditis elegans* (*C. elegans*) showed that 19 chemicals (including phthalates and agrochemicals) increased DNA damage and physiological dysregulation when compared to Bisphenol-A (BPA) [25]. BPA has been widely recognized as a threat to human health, and its use has been highly restricted, with a total ban on its use in infant-related products [26,27]. Due to these concerns, most manufacturers have voluntarily removed BPA from consumer products. With these regulations and demands from consumers, manufacturers must rely on other plasticizers to aid in the performance and functionality of plastic products.

Di-butyl phthalate (DBP), a widely used plasticizer, can compose 20–80% of some plastic products [20] and its use is regulated by both the United States and European agencies [28]. DBP is classified as a phthalate ester, which as a group, are considered endocrine-disrupting chemicals and pose cytotoxic and estrogenic effects [27,29–31]. DBP is most commonly used as a plasticizer, primarily in polyvinyl acetate emulsion adhesives, as a solvent for oil-based dyes, and insecticides [32]. It can be found in consumer products including nail polish, paints, adhesives, and perfume oils. Prior to 2008, many products for children contained high levels of DBP, butyl benzyl phthalate (BBP), and di(2-ethylhexyl) phthalate (DEHP). The United States and European federal restrictions now limit the use of some phthalates, including DBP, in children’s products. Despite bans on some products, DBP and other phthalate esters are widely used in everyday household items or manufacturing processes. Many household products contain these chemical additives, which, when disposed of, will likely end up in municipal landfills [32]. Thus, DBP could leach into the environment due to improper disposal or the breakdown of products containing it. Once in the environment, DBP absorbs onto suspended particles and is less likely to degrade than dissolved DBP [32]. DBP has a high likelihood of being absorbed into MPs in the environment, as it is readily absorbed by plastic particles, possesses properties that make it highly adsorptive, and is chemically related to compounds that are readily absorbed into plastics [33].

Despite the risks of MP exposure to humans and the danger of plastic particles carrying chemical pollutants, MP and chemical co-exposure remain under-investigated. It is crucial to determine how MP–chemical mixtures alter the development and health of humans and other land animals. We use the *C. elegans* model system to examine the cellular and physiological effects of MP mixtures on whole-organism health and development. In this study, we investigate whether 1 μm polystyrene (PS) MPs can transport absorbed chemicals into an animal after ingestion and lead to physiological responses. We chose to work with two different MP doses (1 μg/L and 1 mg/L). These levels of exposure are within the range of other studies and mimic concentrations determined from human samples such as blood [1,34–37]. Polystyrene MP was chosen due to its common use in packing foams, food containers, and disposable cutlery, making it highly relevant to human oral MP exposure risks [20]. The additive DBP was chosen as a representative chemical due to its widespread use as an industrial additive. Studies of additives commonly found in plastics show that many plasticizers with relatively low molecular weight can transfer from plastic materials into foods [20]. We hypothesized that MPs can contain a chemical pollutant and mediate physiological defects that are greater than either pollutant alone. We find that MP exposure reduces brood size and leads to embryonic lethality in a concentration-dependent manner. The MP-mediated delivery further decreased reproduction compared to MPs or DBP alone. Reproductive defects due to MP-DBP co-exposure appear to cause stress via DAF-16 and reduce *C. elegans* lifespan.

## 2. Materials and Methods

### 2.1. Characterization of Microplastics

Polystyrene microplastics (Poly Sciences, 19518-500, Warrington, PA, USA) are purchased in a monodisperse form and contain a slight anionic charge from sulfate ester. PS MP size and surface were characterized by Scanning Electron Microscopy (JEOL JSM-6010 PLUS/LA, Peabody, MA, USA). Samples were sputter-coated with 2 nm gold particles prior to imaging (Quorum Technologies EMS150T ES, Lewes, UK). MP diameter was measured using the measuring tool in the JOEL InTouchScope software (Version 3.01).

### 2.2. Preparation of Microplastic and Chemical Mixtures

MPs were suspended in *Escherichia coli* (strain OP50, grown in LB broth), seeded on Nematode Growth Media (NGM) plates (Teknova, N1105, Hollister, CA, USA) and allowed to dry overnight before adding nematodes to the plates for exposure. To prepare MP *E. coli* suspensions, MPs were suspended in a 1% solution with M9 buffer, then diluted into *E. coli* OP50. At each dilution step, the solution was sonicated for 15 min (Emerson Branson 3800, Danbury, CT, USA). Wide-boar tips were used to transfer liquid. Dry 1 μM PS microspheres (Poly Sciences, 19518-500, Warrington, PA, USA) were used for all experiments unless otherwise noted. Di-butyl phthalate (DBP, Sigma, 524980, Saint Louis, MO, USA) was diluted into 100% DMSO (Sigma, D8418, Saint Louis, MO, USA) at 0.1 M concentration. Additional dilutions were made from the 0.1 M stock into water. DBP PS MP mixtures were prepared by adding DBP (either 0.1 M, 3.7 mM, or 100 μM concentration) to dry PS MPs, to create a 1% solution, which was incubated overnight, on a rotating rack, in the dark, at room temperature (19–22 °C). DMSO PS MP controls were prepared by adding 100% DMSO, 3.7% DMSO, or 0.0001% DMSO to dry PS MPs at 1%. PS MPs were diluted into *E. coli* OP50 to a final concentration of either 1 μg/L or 1 mg/L.

### 2.3. Maintenance of C. elegans

*Caenorhabditis elegans* (*C. elegans*) strains, N2 (wild type) and TJ356 (zIs356 [daf-16p::daf-16a/b::GFP + rol-6(su1006)]) that were used in this study were provided by the Caenorhabditis Genome Center. Worms were grown on Nematode Growth Media Agarose (NGM) plates seeded with *E. coli* OP50 at 20 °C.

### 2.4. Exposure Design

Bleach-synchronized L1 larvae were exposed to *E. coli* OP50 containing PS MPs at either 1 μg/L or 1 mg/L, or PS MPs soaked in DBP (100 μM, 3.7 mM or 0.1 M) or DMSO (0.1%, 3.7%, or 100%) for 24 h, or DBP or DMSO added directly to the OP50 *E. coli* alone on NGM plates made within 48 h. DBP and DMSO-only control concentrations were determined by the amount of solution carried into the final *E. coli* OP50 culture after dilution from the prepared 1% PS MPs. From a 1% PS MP in 0.1 M DBP solution that was diluted 1:10,000 into *E. coli* OP50 for a final concentration of 1 μg/L MP, would have an associated 10 μM DBP control in *E. coli* OP50. For short-term exposure, L1s were removed from PS MPs plates after 48 h, then maintained on prepared *E. coli* OP50 plates until the end points for experimental conditions. At least 2 biological replicates were performed for each assay.

### 2.5. Visualizing Microplastics in C. elegans

To visualize MP distribution and location, L1 bleach-synchronized worms were exposed to 1 μm internally dyed Dragon Green PS microspheres (Bangs Laboratories Inc., FS03F, Fishers, IN, USA). L1 synchronized larvae were exposed to *E. coli* OP50 with 1 μg/L or 1 mg/L Dragon Green MPs suspended in *E. coli* OP50, then seeded on NGM plates. Worms were paralyzed in levamisole on an agarose pad before imaging at each life stage.

### 2.6. Visualizing Nile Red in C. elegans

To assess if chemical compounds absorbed by MP beads could transfer into the worm’s body after ingesting the chemically soaked beads, we fed L1 synchronized *C. elegans* PS MP beads soaked in 1 mg/L Nile Red in methanol (Sigma Aldrich, 19123, Saint Louis, MO, USA). The beads were rocked for 1 h and left to dry overnight, before being suspended in a 1% solution in M9 buffer. The 1% M9 solutions were diluted to 1 μg/L or 1 mg/L in OP50 *E. coli* before seeding on NGM plates and leaving them to dry overnight.

### 2.7. Lifespan Assay

Lifespan assays were performed at 20 °C. Approximately 50 synchronized L1 larvae were placed on the corresponding treatment conditions for 48 h and then transferred to new plates containing the same treatment with 100 μM 5′-fluorodeoxyuridine. Worms were counted as dead or alive every day until the number of live worms reached zero.

### 2.8. Evaluation of Reproductive Toxicity

To assay the total number of eggs laid per worm and the percentage hatched, bleach-synchronized L1 larvae were seeded onto each condition. Then, 48 h after seeding, L4 larval worms were then singled out onto respective plates. The following day, L4 singling is repeated from the previous day’s plate, and both plates are kept. After 24 h, L1s and embryos are counted from the first day of singling, then repeated every day, counting the L4-free plate, until the worm stops laying (around 6–7 days).

### 2.9. Imaging and Microscopy

Fluorescent and bright-field images were all captured using an EVOS M5000 (Invitrogen, Bothell, WA, USA). For live imaging (Dragon-Green PS MP localization, Nile Red-soaked PS MPs transfer, and DAF-16::GFP localization), worms were transferred to an agarose pad on a slide and immobilized with levamisole. All image processing and analysis was performed using ImageJ software (version 1.54).

### 2.10. Activation of Stress Response

To determine if MP and chemical exposure lead to stress activation, the strain TJ356, expressing DAF-16 fused to GFP was exposed to PS MPs alone, and with DBP or DMSO, and DBP and DMSO alone. L1 synchronized larvae were placed onto each condition and live imaged 72 h later. Live imaging was completed within 5 min of paralysis for each strain. Each nematode was classified by the localization of DAF-16::GFP expression (cytoplasmic, intermediate or nuclear) [38,39].

### 2.11. Statistical Analysis

Statistical analysis and figure design were performed with GraphPad Prism 8 (GraphPad Software version 10, San Diego, CA, USA). Data were checked for normality between treatments and statistical analysis was performed using Kruskal–Wallis followed by Dunn’s post hoc test (MP diameter, brood size, PS MP exposure). A *t*-test (two-tailed) was performed to analyze control vs. Nile Red data. Experiments were all carried out with a minimum of 2–3 biological replicates (N) and the number of total worms (n) examined that are used in statistical analysis as described.

## 3. Results and Discussion

### 3.1. Polystyrene Microplastic Surface Structure Is Unchanged After Exposure to Di-butyl Phthalate

**Figure 1.**
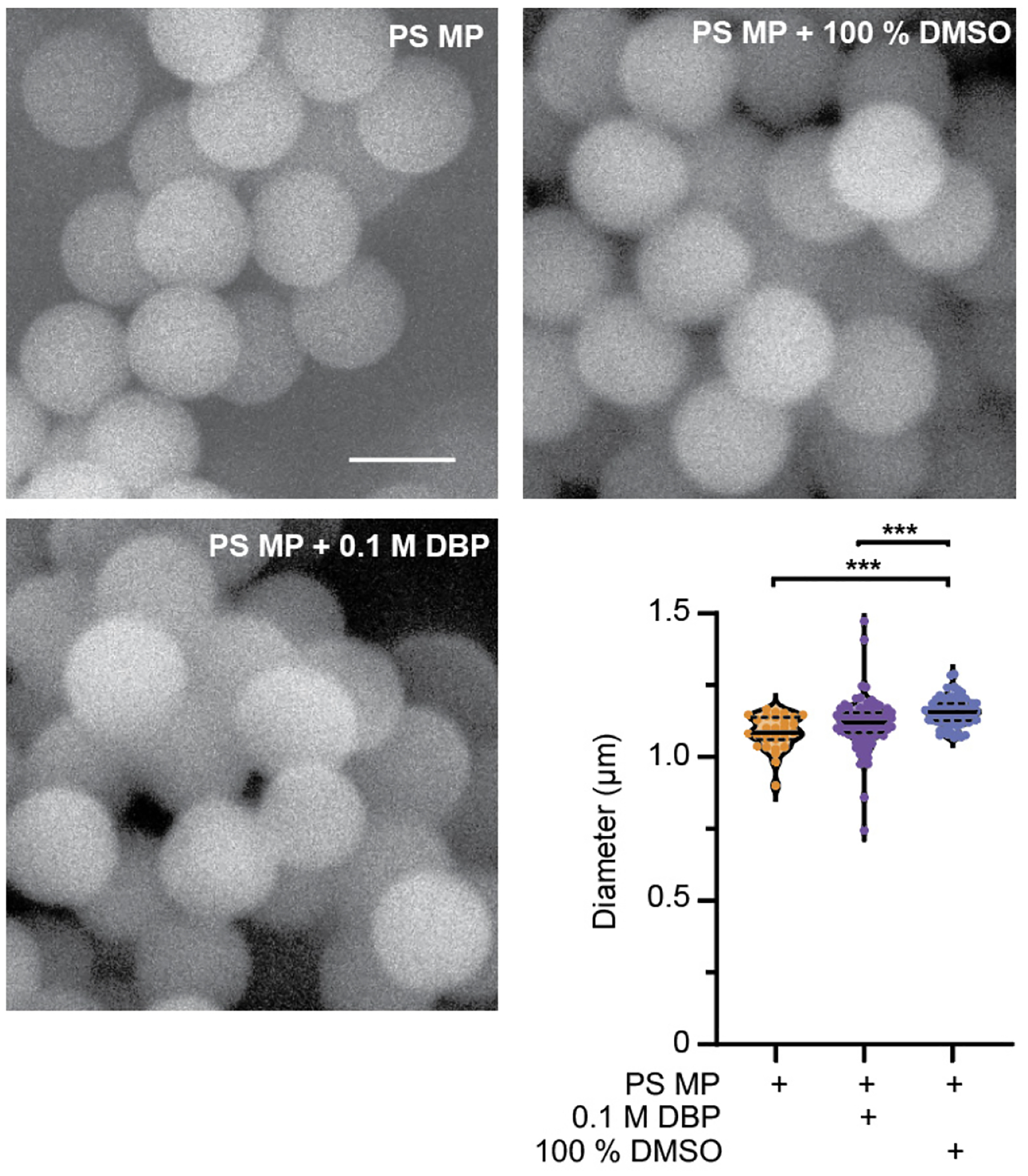
Characterization of polystyrene (PS) microplastics (MP) before and after 24 h exposure to di-butyl phthalate (DBP) and DMSO. Representative SEM images of 1 μm PS MPs to verify their size and surface structure before and after exposure to 0.1 M DBP and 100% DMSO. Scale bar is 1 μm. Quantification of PS MP size before and after 24 h exposure to 0.1 M DBP and 100% DMSO. n = 31, 104, 81, *** *p* ≤ 0.0001.

To verify the diameter and surface structure characteristics of PS microplastics (PS MP, hereon referred to as microplastics), scanning electron microscopy (SEM) was used to image MPs with and without exposure for 24 h to 0.1 M di-butyl phthalate (DBP) or the solvent (DMSO). SEM analysis revealed no change to the surface morphology of MPs exposed to DBP for 24 h. A small but significant increase in the average size of MP particles occurred when soaked in DMSO, but not 0.1 M DBP (Figure 1, *p* ≤ 0.0001). It was anticipated that exposure to the plasticizer DBP may change the size or surface structure of MPs. Environmental exposure of MPs to heat, UV and mechanical stress can change their characteristics [19,40], and this was not observed after 24 h exposure of MPs to DBP or DMSO (Figure 1). DBP has high rates of sorption into MPs, with the highest rates seen in PS MP when compared to polyvinyl chloride (PVC) and polyethylene (PE) [33]. Sorption of compounds on or into MPs is size dependent, with smaller particles containing more readily available chemicals, due to the increase in surface to size ratio that occurs with the reduced particle size [19,41,42]. MP and DBP mixtures were prepared by soaking MPs in DBP for 24 h, followed by dilution into *C. elegans* food, *E. coli* OP50 (in LB). At each dilution step, MPs were sonicated to facilitate resuspension. The sonication process and the addition of MPs, DBP, and DMSO alone or in combination with MPs did not affect the viability of *E. coli* (Figure S1). Additionally, sonication would disrupt any adsorbed DBP on the MP surface, and thus any DBP present in the MP is assumed to be absorbed within the MP particles. The slightly larger, but non-significant, increase in the mean size of the 0.1 M DBP-soaked MPs, and the significantly larger diameter of MPs after DMSO exposure, may be due to swelling of the MP particles after absorption.

### 3.2. Polystyrene Microplastics Are Ingested by C. elegans and Act as a Vehicle for Chemical Exposure

**Figure 2.**
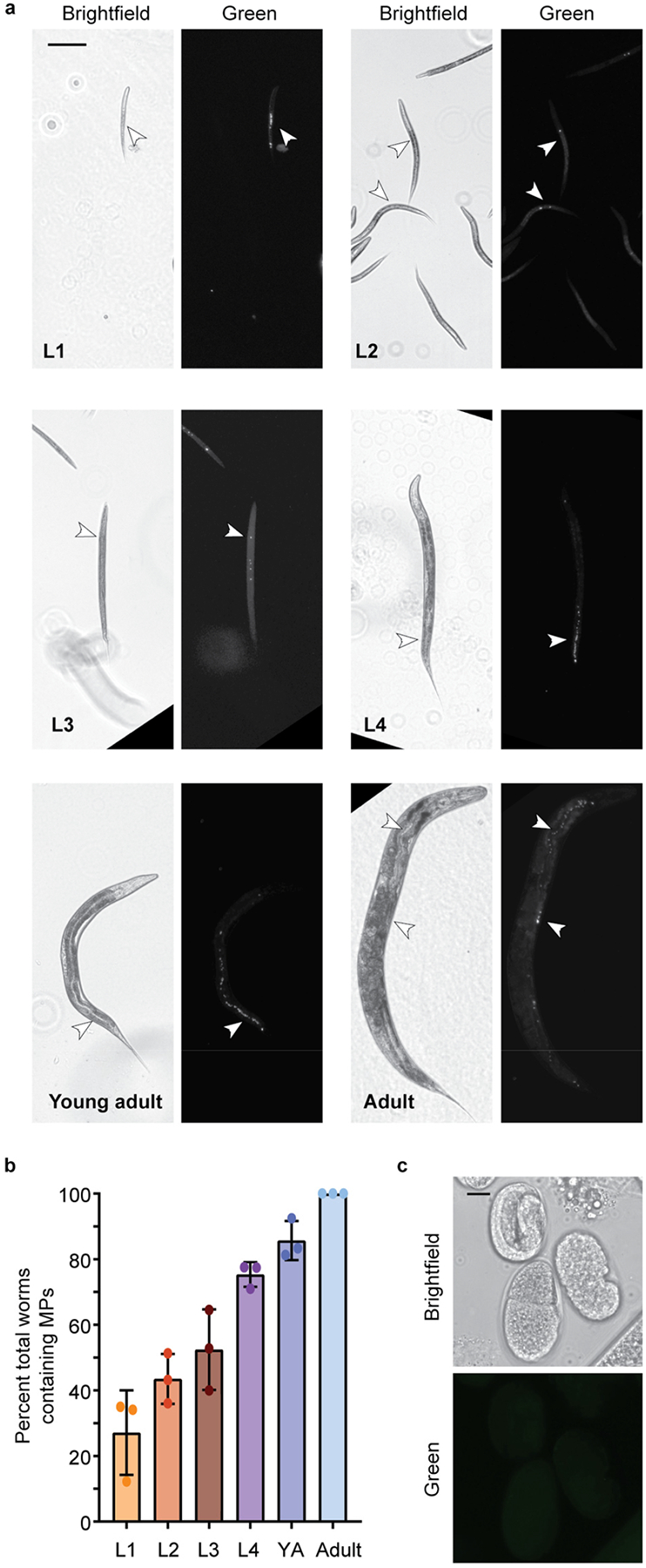
1 μm Dragon Green Polystyrene (PS) Microplastics (MPs) are ingested by *C. elegans*. MPs are ingested by *C. elegans* at all larval stages. (**a**) Representative images of *C. elegans* at life stages, L1–Adult after continuous exposure to 1 μg/L Dragon Green 1 μm PS MP particles. Scale bar = 100 μm. (**b**) Quantification of the number of worms containing MPs at each larval stage (L1–Adult). YA, young adult. N = 3, n = 173, 247, 199, 111, 111, 105. (**c**) Representative image of *C. elegans* eggs from an adult worm that was continuously exposed to Dragon Green PS MPs. Scale bar = 10 μm.

*C. elegans* exposed to 1 μg/L and 1 mg/L MP show the presence of MPs in their gut tube (Figures 2a and S2). Nematodes were initially exposed to two doses (1 μg/L and 1 mg/L), within the range of other studies and mimicking concentrations determined in human samples, such as blood [1,34–37]. MPs were present at all larval life stages, and the percentage of total worms containing MPs increased with life stage and exposure time. By adulthood, nearly all worms contained MPs (Figure 2b). These results are consistent with other studies demonstrating that MPs (>1 μm) accumulate in the gut, while smaller NP (<1 μm) particles can cross cell membranes [14,36,43–45]. Smaller MPs, less than 1 μm in size, in the nanometer range, have been observed to cross into the body cavity in *C. elegans* [43]. However, when examining the eggs of adult nematodes exposed to Dragon-Green MP, no MPs were observed (Figure 2c).

**Figure 3.**
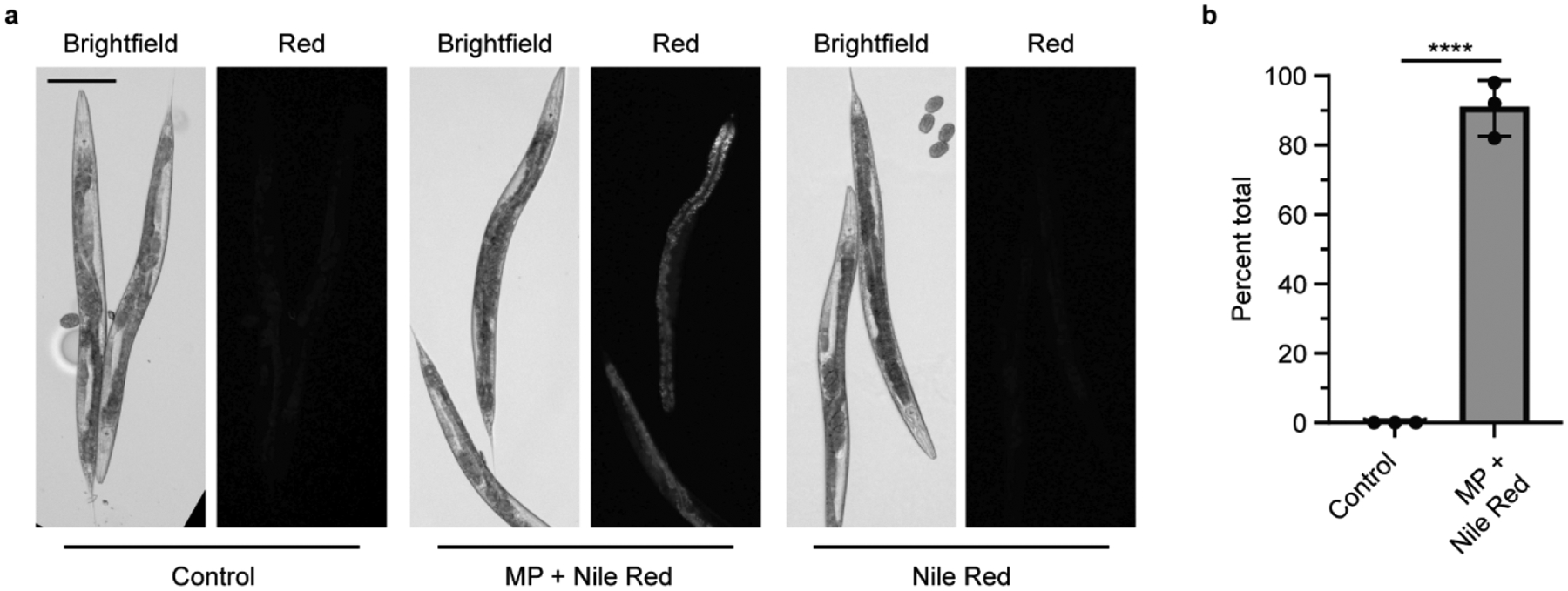
Microplastics soaked in Nile Red act as a carrier to deliver Nile Red into *C. elegans* after ingestion. (**a**) Representative images of adult *C. elegans* fed *E. coli* OP50 with and without Nile Red-stained microplastics (MPs). Control = *E. coli* OP50 only. Scale bar = 100 μm. (**b**) Quantification of the presence of Nile Red inside *C. elegans* after ingestion of Nile Red-stained MPs, N = 3, n = 50, 50, 30. **** *p* < 0.00001.

MPs contain chemical additives or may absorb pollutants in the environment that can then be transferred into living organisms, leading to physiological effects [20,33]. Phthalates, including DBP, are classified as endocrine disruptors that can be long-lived in the environment [20,41]. DBP is most commonly used as a plasticizer in PVC, followed by polyethylene terephthalate (PET) [20]. While DBP is not commonly used in PS products, we chose to study it as a potential environmental chemical that could be absorbed in MPs and transferred into an organism after ingestion. To visualize this, we soaked PS MPs in the lipophilic dye Nile Red to determine whether MPs could mediate the delivery of a chemical into an organism. Nematodes exposed to Nile Red-soaked MPs do not show puncta, which indicate the location of each MP, but rather show diffuse staining in the worm (Figures 3a and S3). This indicates that the Nile Red dye absorbed by the PS MP, transfers into the worm’s body after ingestion. This transfer is present in more than 90% of the worms examined (Figure 3b). When nematodes are exposed to an equivalent amount of Nile Red without MPs, the Nile Red is not present in the worm’s body. Thus, the MPs mediate the delivery of Nile Red, which is then absorbed. This mimics what one would expect if MPs can act as a vector to deliver chemicals into an organism after ingestion.

### 3.3. Exposure to Polystyrene Microplastics and Polystyrene Microplastic DBP Mixtures Results in Reproductive Toxicity

**Figure 4.**
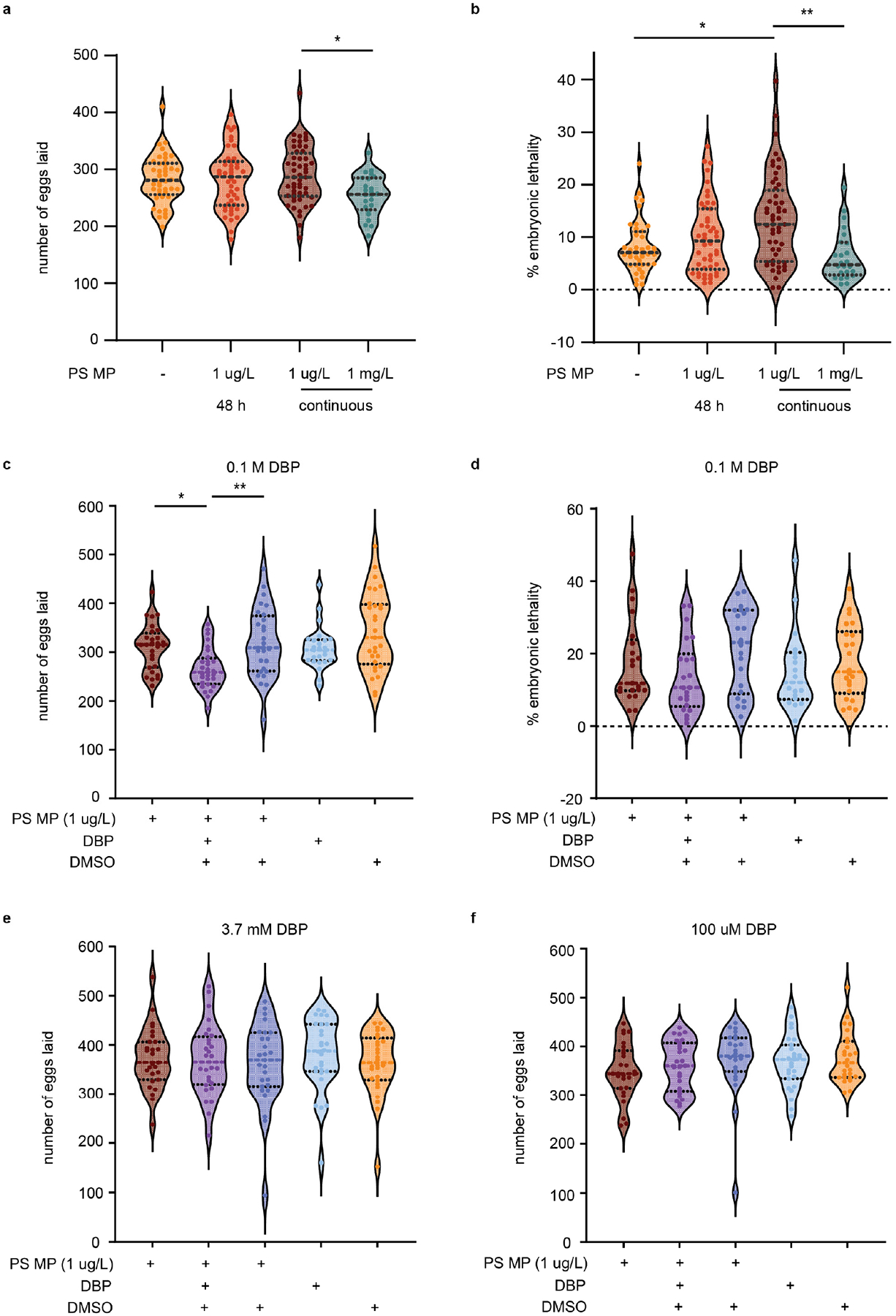
Total eggs laid, number of fertilized and unfertilized eggs, and embryonic lethality of *C. elegans* exposed to polystyrene (PS) microplastics (MP) and/or containing DBP. (**a**) Number of eggs laid per nematode with *E. coli* OP50 only, 48 h exposure to PS MP or continuous PS MP exposure. N = 8, n = 43, 46, 52. (**b**) Percent embryonic lethality. (**c**) Total number of eggs with exposure to continuous PS MP with and without 0.1 M DBP. N = 4, n = 28, 28, 28, 25, 29. (**d**) Percent embryonic lethality. (**e**) Total number of eggs with exposure to continuous PS MP with and without 3.7 mM DBP. N = 4, n = 27, 28, 27, 28, 28. (**f**) Total number of eggs with exposure to continuous PS MP with and without 100 μM DBP. N = 3, n = 27, 27, 25, 27, 26. Violin plots show the mean with the standard deviation. Kruskal–Wallis was used for statistical analysis, using Dunn’s as a post hoc test (* *p* ≤ 0.05; ** *p* ≤ 0.01).

To determine if MPs and chemical pollutants cause reproductive toxicity, *C. elegans* were exposed to different concentrations of MPs and DBP for varying times. When L1 life stage, nematodes were exposed to 1 μm MP (at 1 μg/L concentration) for 48 h, until the young adult stage, or continuously, there was no observed significance in decreased total eggs laid or hatched (Figures 4a and S4a). Despite no change to the number of eggs laid, chronic MP exposure to nematodes significantly increased embryonic lethality (*p* = 0.04) and the total number of eggs unhatched per nematode (*p* = 0.007) (Figures 4b and S4b). Embryonic lethality is determined by taking the total number of eggs that are unhatched and dividing by the total number of eggs laid per worm. These results are within the range of other studies that observed continuous MPs of the same or smaller size, leading to minimal or no change in brood size and no embryonic lethality [45]. While some studies reported a decrease in brood size with exposure to MPs, this difference may be due to differences in MP size or composition or to varying exposure conditions [46]. When we exposed *C. elegans* to a 1000-fold higher concentration of MPs (1 mg/L), we observed a significant reduction in the total number of eggs laid (*p* = 0.016); however, this did not lead to an increase in embryonic lethality (Figure 4a,b).

*C. elegans* exposure to MPs (1 μg/L) harboring 0.1 M DBP further reduced the number of eggs laid per worm compared to exposure to MPs alone (*p* = 0.017, Figure 4c). This combined exposure did not increase embryonic lethality (Figure 4d). Examination of MPs loaded with either 100 μM or 3.7 mM DBP did not affect brood size, egg hatching, or embryonic lethality (Figures 4e,f and S5a–f). Typical concentrations of DBP used as a plasticizer can range from 10–35%, or higher in some products [20]. Our study employed 0.1 M DBP, a concentration equivalent to less than 10% *w*/*w*, which is in the range of DBP found in everyday products [20]. While 0.1 M DBP caused defects in fertility, these were not observed at lower concentrations (100 μM and 3.7 mM). Comparable levels of chemical pollutants could reach organisms through the ingestion of MPs that have absorbed chemicals from the environment or by the leaching of additives from MPs that initially contained higher chemical amounts. In particular, it is worth noting that recycled plastics can contain additives from recycled feedstocks [47].

Impaired reproduction with exposure to MPs alone or in combination with DBP could be caused by various factors. DBP is an endocrine-disrupting chemical and leads to cryptogenic effects [31]. Exposure to DBP leads to defects in reproduction and development [28]. Specifically, DBP exposure reduced human sperm function and in rodent studies defects occur in both development and reproduction [48–51]. In *C. elegans*, exposure to 100 μM DBP, in the absence of MP, led to increased embryonic lethality and DNA damage [25]. *C. elegans* exposure to DBP alone, at a lower dose (500 μM) than used in this study, leads to a reduction in the number of eggs laid and increased embryonic lethality [25].

These defects were attributed to defects in early embryogenesis, with elevated levels of DNA double-strand breaks, activation of a DNA damage checkpoint, and impaired embryogenesis [25]. Errors in cell division could reduce the ability of eggs to hatch and could be caused by a multitude of sources [52]. In humans and other organisms, these errors may lead to aneuploidy, spontaneous abortions, and birth defects [25,52,53]. Additionally, a reduction in the total number of eggs laid could be attributed to a nutrient deficiency of the parent caused by intestinal damage from PS MP exposure [46,54–57]. Another study suggested that the associated defects with PS MP exposure could be due to a reduction in ATP levels and a reduced energy budget toward reproduction [58].

The exacerbated defects we observed with co-exposure to MPs containing 0.1 M DBP demonstrate that MPs can mediate the delivery of a chemical into an organism after ingestion. This MP-mediated delivery of DBP underscores the risks of co-exposure when MPs can release the additives they already contain and/or chemicals that they have absorbed from the environment.

### 3.4. Exposure to Polystyrene Microplastics and Polystyrene Microplastic DBP Mixtures Elicits a Stress Response

A possible mechanism of PS MP toxicity resulting in a reduction in fertility is the generation of reactive oxygen species (ROS). PS MP exposure leads to higher levels of reactive oxygen species [15,35,54] and increased expression of GST-4 (glutathione S-transferase 4, an enzyme that is involved in clearing ROS) in nematodes [45,56]. Additionally, DBP and other phthalate esters cause DNA damage and chromosomal abnormalities [25,53] that can be caused by ROS generation [51]. In response to stress, *C. elegans* activate a DAF-16 stress response that leads to the activation of genes encoding proteins involved in response to oxidative stress [59]. Therefore, to investigate if stress may be an underlying mechanism of reduced fertility, we asked if MP-mediated DBP exposure in *C. elegans* would initiate a DAF-16 stress response. The *C. elegans* strain TJ356 contains a reporter GFP fused to DAF-16 that is driven by the daf-16 promoter [60]. This GFP reporter is cytoplasmic under normal conditions and relocates to the nucleus to activate genes involved in an anti-oxidative response, under stress. Therefore, the localization of this DAF-16 GFP reporter can be used as a readout of a DAF-16-mediated stress response [35,38,45].

**Figure 5.**
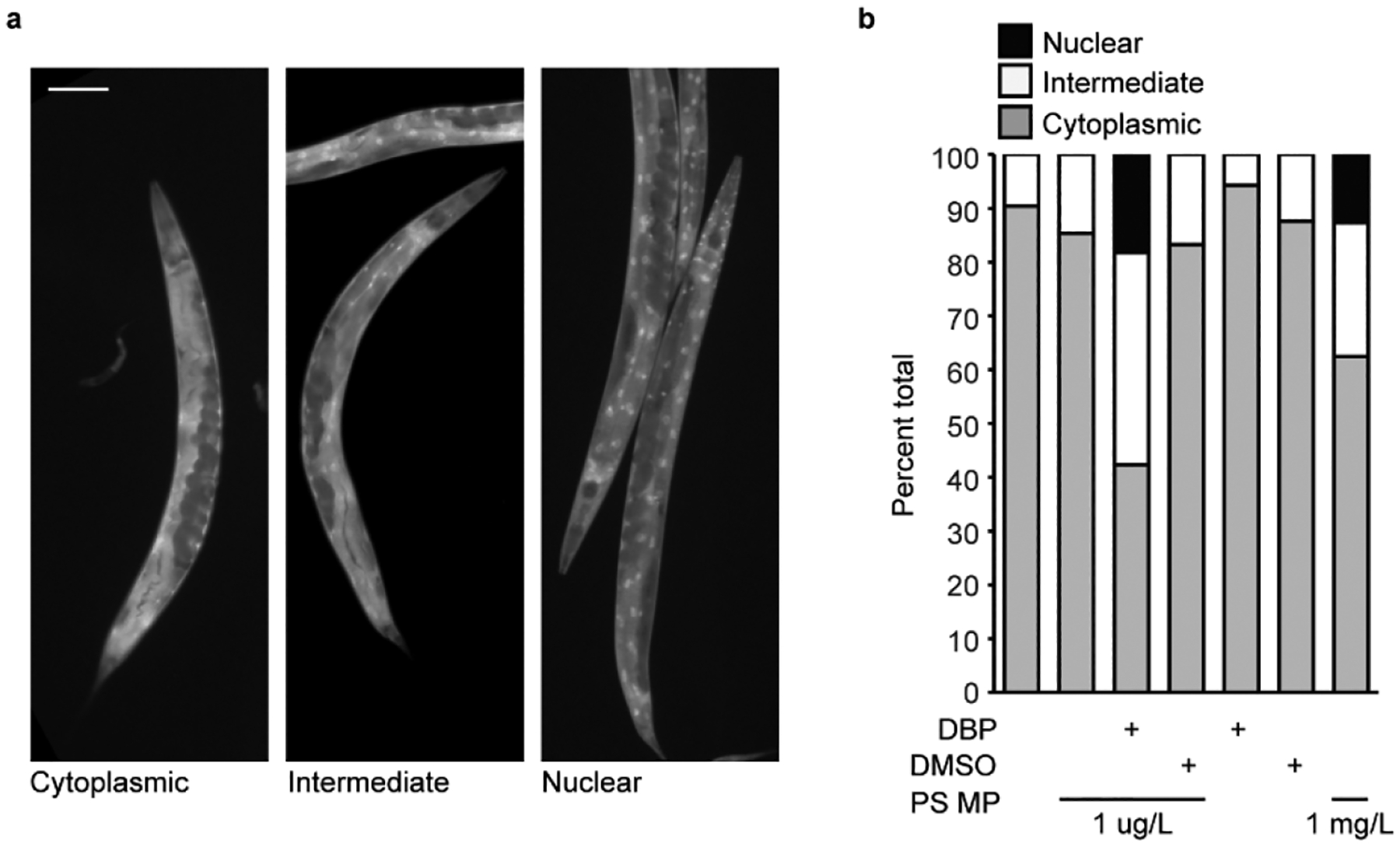
Stress response via DAF-16 in *C. elegans* after exposure to polystyrene (PS) microplastic (MPs) and DBP. (**a**) Representative images of DAF-16-GFP expression in adult *C. elegans*, showing cytoplasmic (left), intermediary (center), and nuclear localization (right). Scale bar = 100 μm. (**b**) Quantification of DAF-16-GFP localization in *C. elegans* with exposure to their regular *E. coli* diet only, with 1 μg/L PS MP, 1 μg/L PS MP and DBP, 1 μg/L PS MP and DMSO (vehicle control), DBP alone, and DMSO alone. N = 2, n = 53, 65, 170, 149, 140, 129, 43.

Under control conditions, when *C. elegans* were not exposed to MPs, majority of the worms examined had cytoplasmic DAF-16 localization (Figure 5a,b). Some worms showed an intermediate localization where less than 30% of the nuclei showed nuclear expression. This same expression pattern is present with *C. elegans* exposure to 1 μg/L MPs (Figure 5b). These results are consistent with what others see with similar PS MP exposure [45]. In contrast, MP-mediated DBP exposure led to a significant activation of the DAF-16 stress response, with 40% of the total worms showing an intermediate expression pattern and nearly 20% nuclear localization (Figure 5b). This indicates that the DAF-16 transcription factor is located in the nucleus, where it will activate genes in response to oxidative stress. Importantly, exposure to MPs containing DMSO, the solvent for DBP, or an equivalent amount of DBP or DMSO alone did not affect DAF-16 localization (Figure 5b). Expression of DAF-16 in these controls showed no significant change compared with the negative control (*E. coli* OP50 only) or MP exposure alone (Figure 5b). In our initial reproductive toxicity studies, we observed that higher concentrations of MPs (1 mg/L) induced a reduction in total eggs laid with no effect on embryonic lethality (Figure 4a,b). Therefore, we asked if MPs alone, at a higher concentration, would elicit a stress response via DAF-16. Continuous exposure of *C. elegans* to 1 mg/L MPs increased the percentage of worms with intermediate and nuclear expression of DAF-16 (Figure 5b). This indicates that the concentration of MP exposure can mitigate the effect. In contrast to our results, Leon et al. observed a stress response with *C. elegans* exposure to 100 nm PS MPs at a concentration of 10 mg/L [45]. This may be due to differences in the size of the tested plastic particles. The stress response observed in Leon et al. was with MPs of the same composition, PS, but with a smaller diameter (100 nm) and at 10,000-fold greater concentration than in this study. Indeed, we observed that 1 μm MPs at our higher, 1 mg/L, concentration elicited a DAF-16 stress response. This indicates that a stress response via DAF-16 may be concentration-dependent. While we did not see a DAF-16-mediated stress response with our primary level of MP exposure (1 μM/L) alone, a significant response was elicited when DBP delivery was mediated with this same concentration of MPs. Interestingly, Leon et al. observed that with the removal of worms from MP exposure, the MPs left the worm’s body, and the DAF-16 stress response reduced [45]. We did not test for the effect of DAF-16 localization with the removal of MP exposure from *C. elegans*, but it would be interesting to ask if the MP-mediated DBP stress response via DAF-16 would be maintained, as the MPs might clear the worm body, while the DBP is absorbed.

### 3.5. Exposure to Polystyrene Microplastics and Microplastic DBP Mixtures Reduces Lifespan

**Figure 6.**
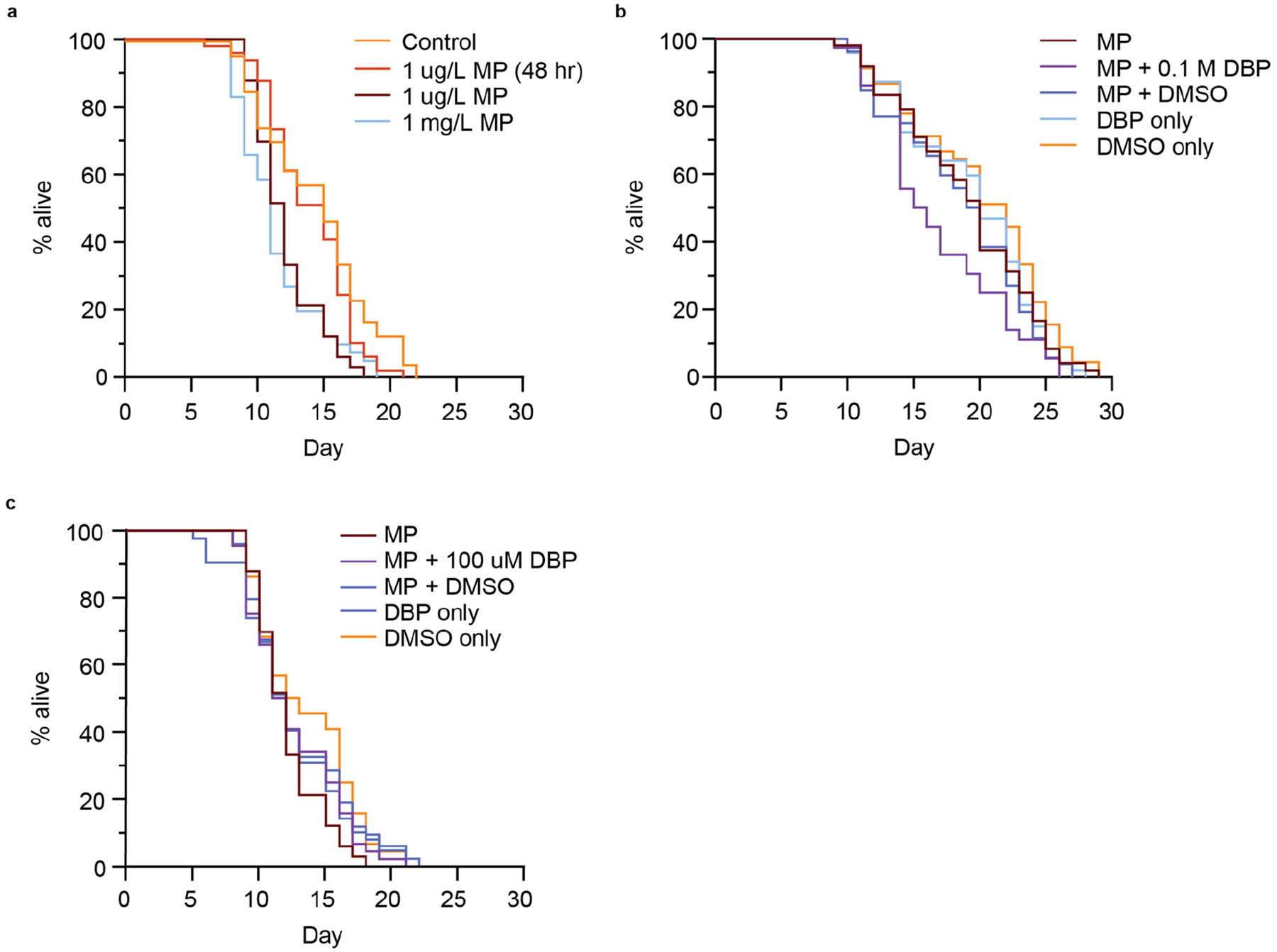
Lifespan of *C. elegans* exposed to polystyrene (PS) microplastic (MPs) containing different concentrations of MPs or MPs that had been soaked in different concentrations of DBP (0.1 M or 100 μM). (**a**) Survival assay for nematodes exposed to control (no MPs), 1 μg/L PS-MPs for 48 h, 1 μg/L or 1 mg/L PS-MPs continuously. n = 47, 49, 33, 41. *p* < 0.0001. (**b**) Survival assay for N2 nematodes continuously exposed to 1 μg/L PS MPs, with and without being soaked in 0.1 M DBP or vehicle alone (100% DMSO) for 24 h. n = 48, 36, 52, 47, 45. *p* = 0.00357. (**c**) Survival assay for N2 nematodes continuously exposed to 1 μg/L PS-MPs, with and without being soaked in 100 μM DBP or vehicle alone (0.1% DMSO) for 24 h. n = 33, 44, 42, 49, 44. Not significant, *p* = 0.4. Log-rank (Mantel–Cox).

In some cases, the activation of a DAF-16 stress response is associated with an extended lifespan [39,59,61,62], and this response can be tissue-specific, with greater extension of lifespan associated with intestinal-specific DAF-16 activation [63]. Increased longevity induced by stress events is associated with the insulin/insulin-like growth factor (IGF-1) receptor signaling pathway, or diet, amongst other pathways [62]. In particular, due to the presence of MP accumulation in the gut of the worm (Figure 2a), and studies indicating intestine-specific stress due to MP exposure [57], we wondered if the presence of MPs may reduce nutrient intake and increase lifespan. We next addressed whether MPs or MP-mediated DBP exposure would alter *C. elegans* lifespan. Short-term exposure (48 h) of *C. elegans* to 1 μm MP (1 μg/L) did not change the lifespan of animals compared to the control (Figure 6a). Interestingly, when nematodes were exposed to the same conditions, but without removal after 48 h, there was a significant reduction in lifespan (*p* < 0.0001) (Figures 6a and S6a,b). This reduction in lifespan was equal to what was observed with continuous exposure to 1000-fold higher concentrated MPs (1 mg/L) (Figure 6a). The lifespan of animals was further reduced when exposed to MPs that mediated the delivery of 0.1 M DBP (*p* = 0.00357) (Figures 6b and S6c). This reduction in lifespan was not observed under the control conditions of MPs containing DMSO, or with exposure to DBP or DMSO alone (Figures 6b and S6c). Additionally, the reduction in lifespan with MP-mediated DBP exposure appears to be dependent on the concentration of DBP, at the lowest concentration used in this study (100 μM), did not further reduce lifespan compared to MP exposure alone (Figures 6c and S6c). There are conflicting studies on the effect of MP exposure on *C. elegans* lifespan. In one study, PS MPs initiated a DAF-16 stress response that was not associated with a change in lifespan in *C. elegans*, and this was speculated to be due to a counteracting mechanism [45]. Other reports were consistent with our results, showing reduced lifespan with exposure to PS MPs [35]. Furthermore, our work shows that MP-mediated delivery of DBP further reduced lifespan. Additional reports examined the effects of leachates from plastic [64], phthalates [65,66], and other types of MP exposure on lifespan, all leading to reduced longevity.

This work demonstrates that MP-mediated delivery of plastic-containing compounds, such as DBP, enhances the physiological defects observed with MP exposure alone. The exposure level of MPs, the time of exposure, and the concentration of the absorbed chemicals influence the defects observed. This provides evidence that *C. elegans*, like other model organisms, can serve to assess the risk associated with microplastic-facilitated exposure to chemical compounds [67,68]. There is a need for whole-animal model organism systems to systematically interrogate the synergistic effects of co-exposure to microplastics and other chemical compounds (e.g., chemical additives, environmental toxins, metals, antibiotics, etc.). Systematic, rigorous whole-animal model studies will make it possible to understand long-term and multigenerational effects of these pollutants.

Overall, our results show that chronic MP exposure has detrimental effects on reproduction and reduces lifespan. Specifically, we demonstrate that co-exposure of MPs and DBP exacerbates the defects observed. MPs mediate DBP delivery in *C. elegans*, further decreasing fertility and lifespan and triggering a stress response via DAF-16 activation. Further studies are needed to identify other mechanisms underlying the toxicity associated with MP-mediated exposure to DBP. The worsened physiological defects seen with co-exposure to phthalate-containing MPs shows that MPs can leach contents into an organism after ingestion. This highlights the risks of MPs releasing the additives they already contain and/or chemicals that they have absorbed from the environment.

## Supplementary Material

Supplementary Information

**Supplementary Materials:** The following supporting information can be downloaded at https://www.mdpi.com/article/10.3390/microplastics4040096/s1. Figure S1: Viability of *E. coli* OP50 when sonicated, or with the addition of MPs, DBP, DMSO alone, or in combination; Figure S2: Schematic of the experiments to visualize microplastics (MPs) in *C. elegans*; Figure S3: Schematic of reproductive and physiological stress assays using various *C. elegans* strains; Figure S4: Number of eggs hatched and unhatched in *C. elegans* exposed to 48 h or continuous polystyrene (PS) microplastics (MPs) with and without DBP; Figure S5: Number of eggs hatched, unhatched eggs, and embryonic lethality of *C. elegans* exposed to polystyrene (PS) microplastic (MP) that had been soaked in different concentrations of DBP; Figure S6: Lifespan of *C. elegans* exposed to polystyrene (PS) microplastic (MP) and 0.1 M DBP.

## Data Availability

The raw data supporting the conclusions of this article will be made available by the authors on request.

## Abbreviations

The following abbreviations are used in this manuscript:

MP: Microplastic
NP: Nanoplastic
C. elegans: Caenorhabditis elegans
BPA: Bisphenol A
DBP: Dibutyl phthalate
BBP: Butyl benzyl phthalate
DEHP: Di(2-ethylhexyl) phthalate
DMSO: dimethyl sulfoxide
E. coli: Escherichia coli
NGM: Nematode Growth Media Agarose
L1: Larval stage 1
L2: Larval stage 2
L3: Larval stage 3
L4: Larval stage 4
GFP: Green fluorescent protein
PS: Polystyrene
SEM: Scanning electron microscopy
PVC: Polyvinyl chloride
PE: Polyethylene
YA: Young adult
ROS: Reactive oxygen species
GST-4: Glutathione S-transferase 4
DAF-16: Forkhead box O (FOXO) protein
IGF-1: Insulin/insulin-like growth factor
