# Supplementary material for: Microplastic-Mediated Delivery of Di-butyl Phthalate Alters C. elegans Lifespan and Reproductive Fidelity
Source: Microplastics. Author manuscript; Available in PMC 2026 Feb 11. (PMC12889887; doi:10.3390/microplastics4040096)
Supplement: Supplementary Information [file NIHMS2140016-supplement-Supplementary_Information.pdf]

Maldonado et al

Supplemental Figures

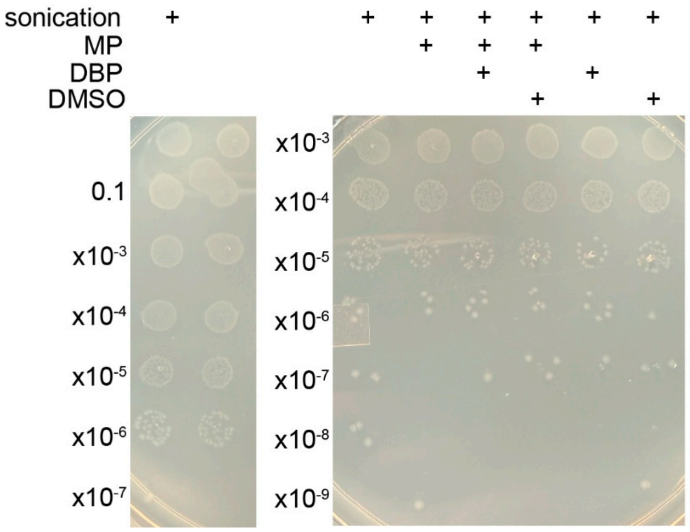

Supplemental Figure S1: Viability of *E. coli* OP50 when sonicated, or with the addition of MPs, DBP, DMSO alone, or in combination. All conditions were diluted into *E. coli* OP50 in LB broth, then sonicated for 15 minutes. Suspensions were immediately plated onto NGM plates and dried overnight.

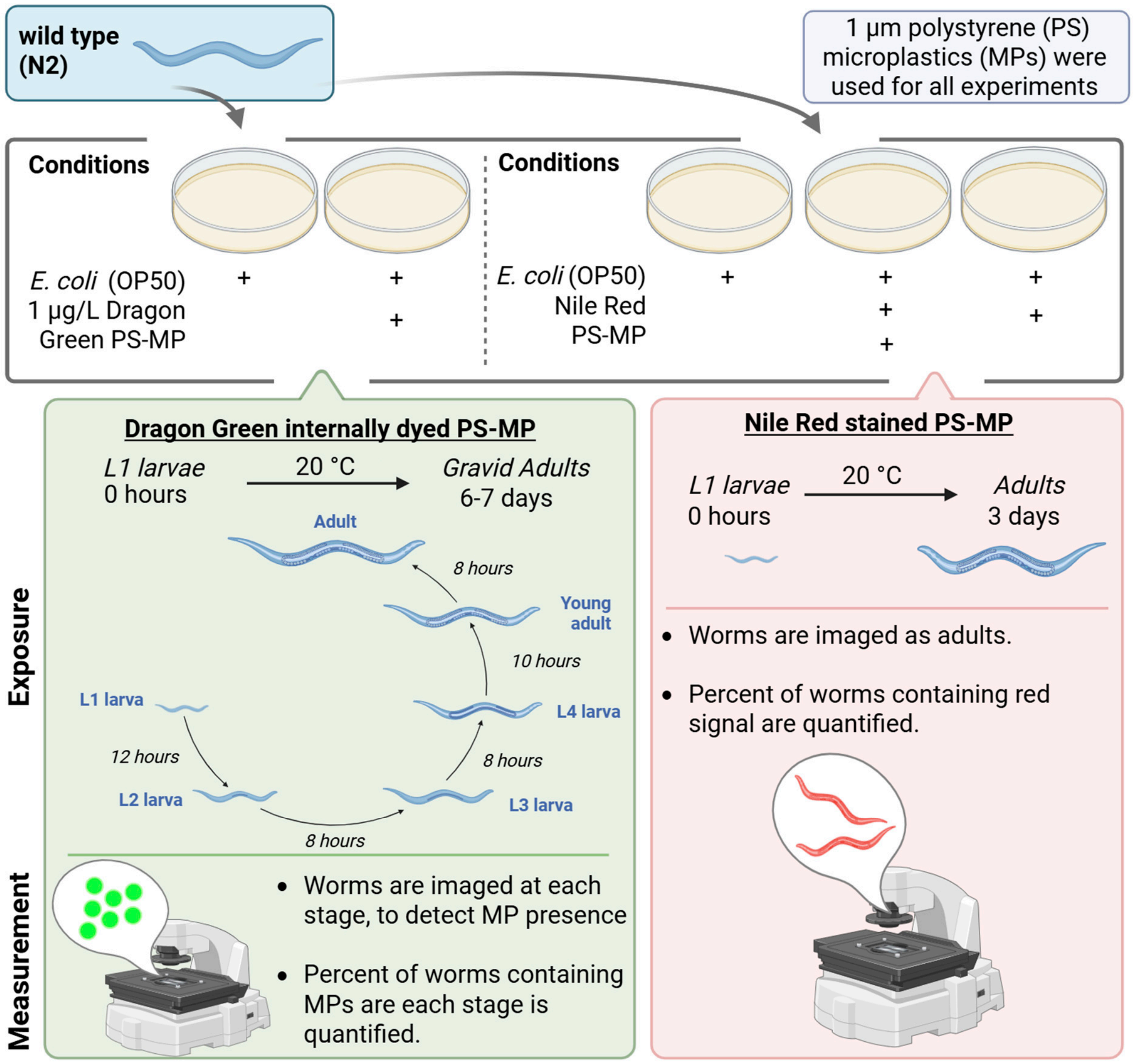

Supplemental Figure S2: Schematic of the experiments to visualize microplastics (MPs) in *C. elegans* after exposure to Dragon Green and Nile Red polystyrene (PS) microplastics (MPs). Wild type N2 nematodes are exposed to 1 µg/L GFP PS MPs, then imaged at each life stage to detect MP accumulation. Wild type N2 nematodes are also exposed to 1 mg/L Nile Red-soaked PS MPs, then imaged as adults to observe chemical transfers from the microplastic to the *C. elegans*. Created with BioRender.com.

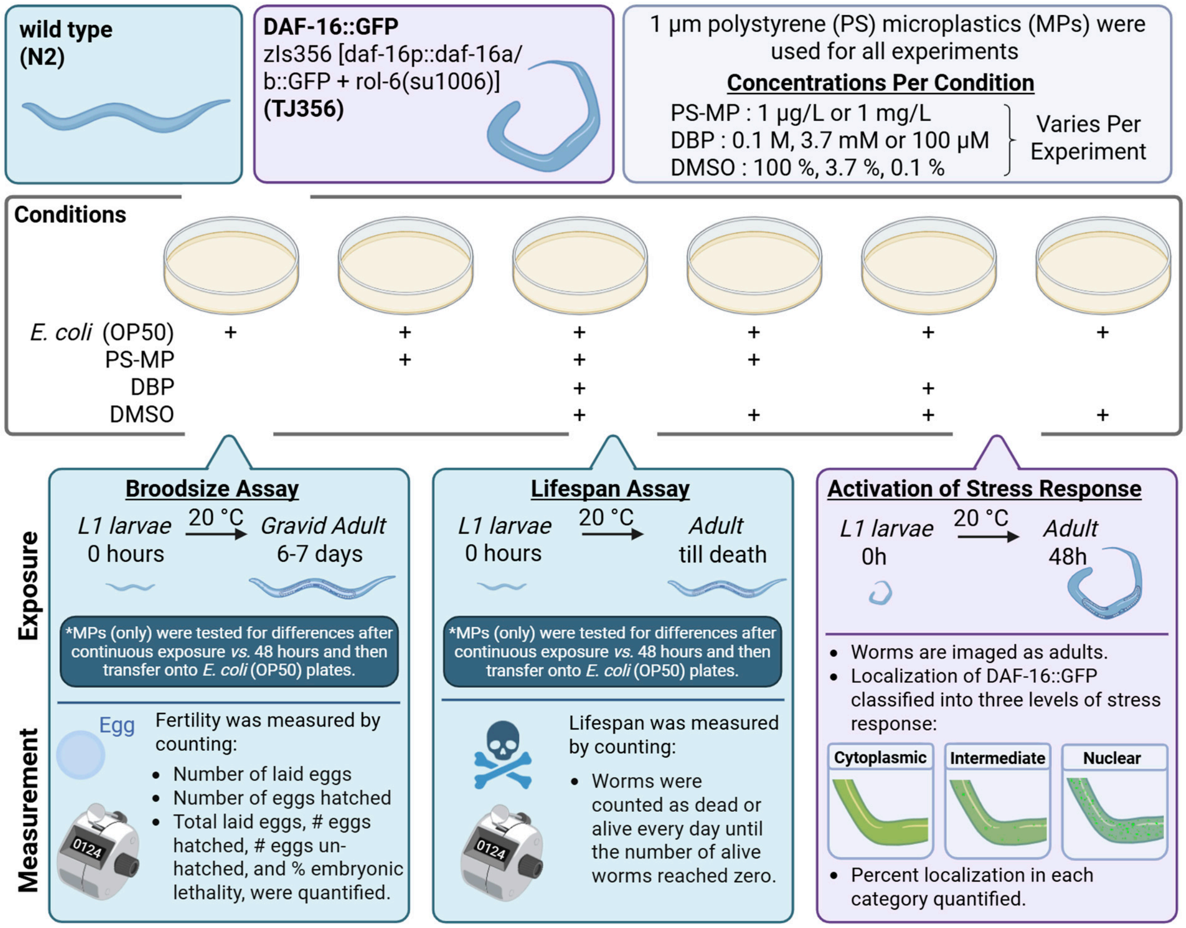

Supplemental Figure S3: Schematic of reproductive and physiological stress assays using various *C. elegans* strains. Wild type N2 nematodes were used for brood-size assays to determine fertility by counting the number of eggs laid versus the number hatched. Wild type N2 nematodes were used in lifespan assays to determine the rate of survival per condition from L1 larvae stage to adults. The DAF-16::GFP containing strain (TJ356) allows for visualization of the DAF-16 transcription factor that relates to the nucleus under stress. Created with BioRender.com.

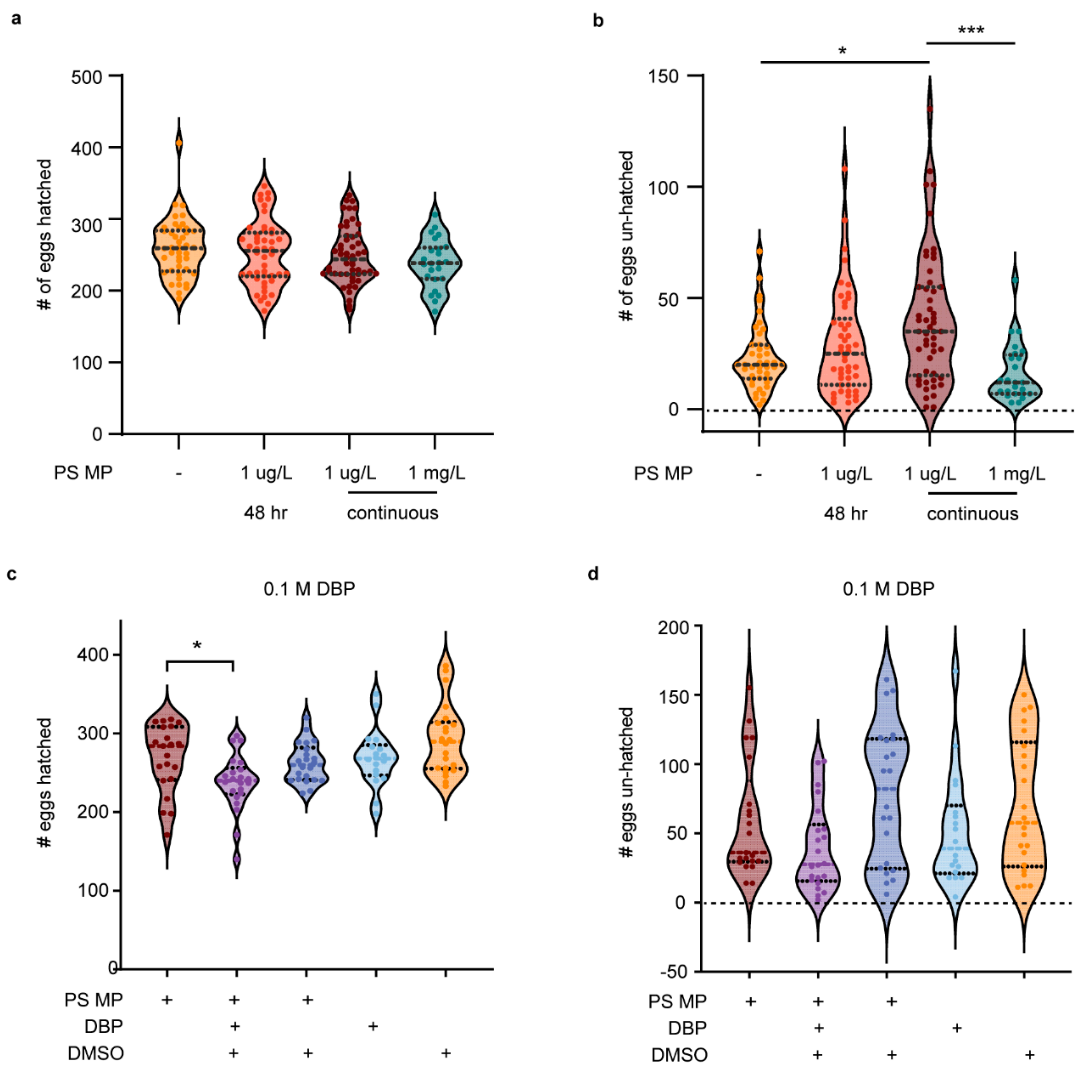

Supplemental Figure S4: Number of eggs hatched and unhatched in *C. elegans* exposed to 48 hours or continuous polystyrene (PS) microplastics (MPs) with and without DBP. a) Number of eggs hatched with exposure to *E. coli* OP50 only, 48-hour exposure to PS MPs, or continuous PS MP exposure. N = 8, n = 43, 46, 52. b) Number of eggs unhatched. c) Percent eggs hatched. d) Number of eggs hatched with exposure to continuous PS MP with and without 0.1 M DBP. N = 4, n = 27, 28, 27, 28, 28. Violin plots show the mean with the standard deviation. Kruskal-Wallis was used for statistical analysis, using Dunn's as a post hoc test (*$p \leq 0.05$; ***$p \leq 0.001$).

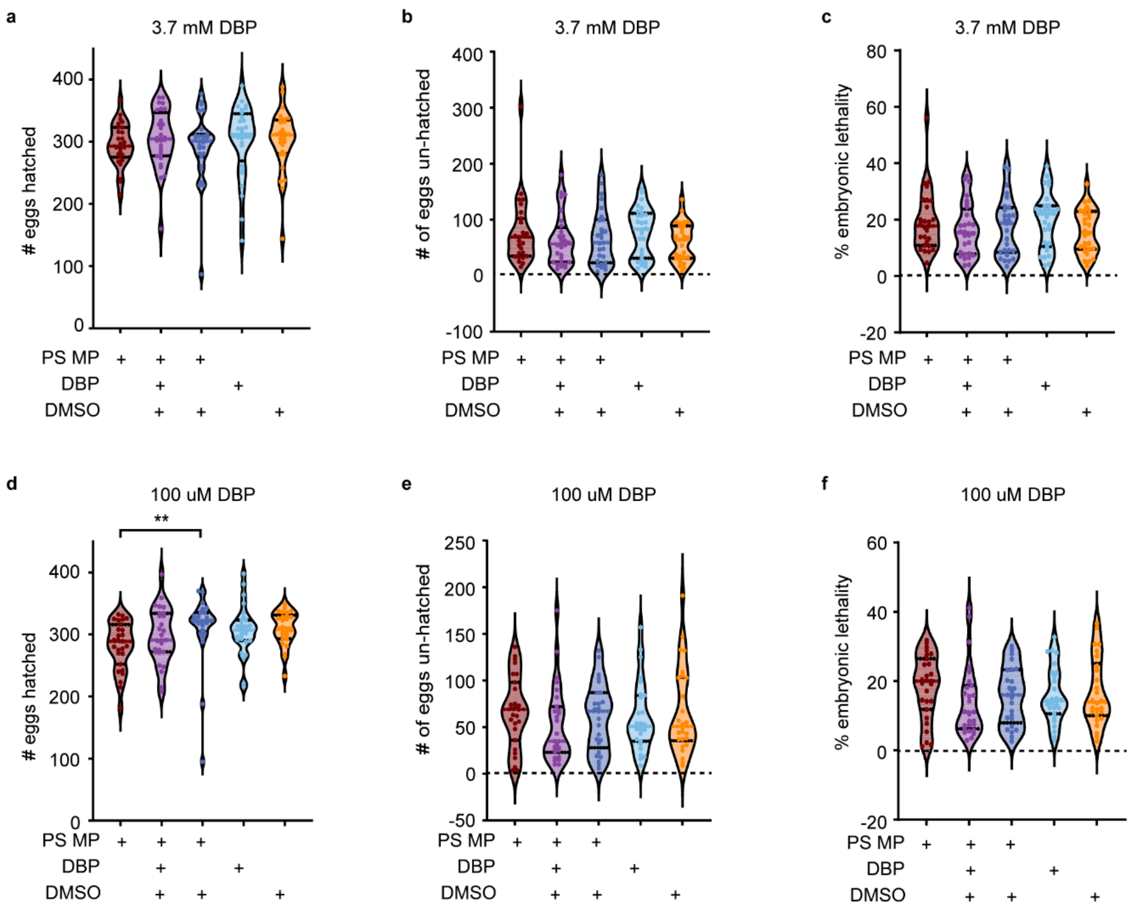

Supplemental Figure S5: Number of eggs hatched, unhatched eggs, and embryonic lethality of *C. elegans* exposed to polystyrene (PS) microplastics (MPs) soaked in different concentrations of DBP (3.7 mM or 100 μM). a-c) Nematode exposure to continuous PS MP with and without 3.7 mM DBP. N = 4, n = 27, 28, 27, 28, 28. a) Number of eggs hatched, b) Number of eggs unhatched, c) Percent embryonic lethality. d-f) Nematode exposure to continuous PS MPs with and without 100 μM DBP. N = 3, n = 27, 27, 25, 27, 26. d) Number of eggs hatched, e) Number of eggs unhatched, f) Percent embryonic lethality. Violin plots show the mean with standard deviation. Kruskal-Wallis was used for statistical analysis, using Dunn's as a post hoc test, $^{**}p \leq 0.01$.

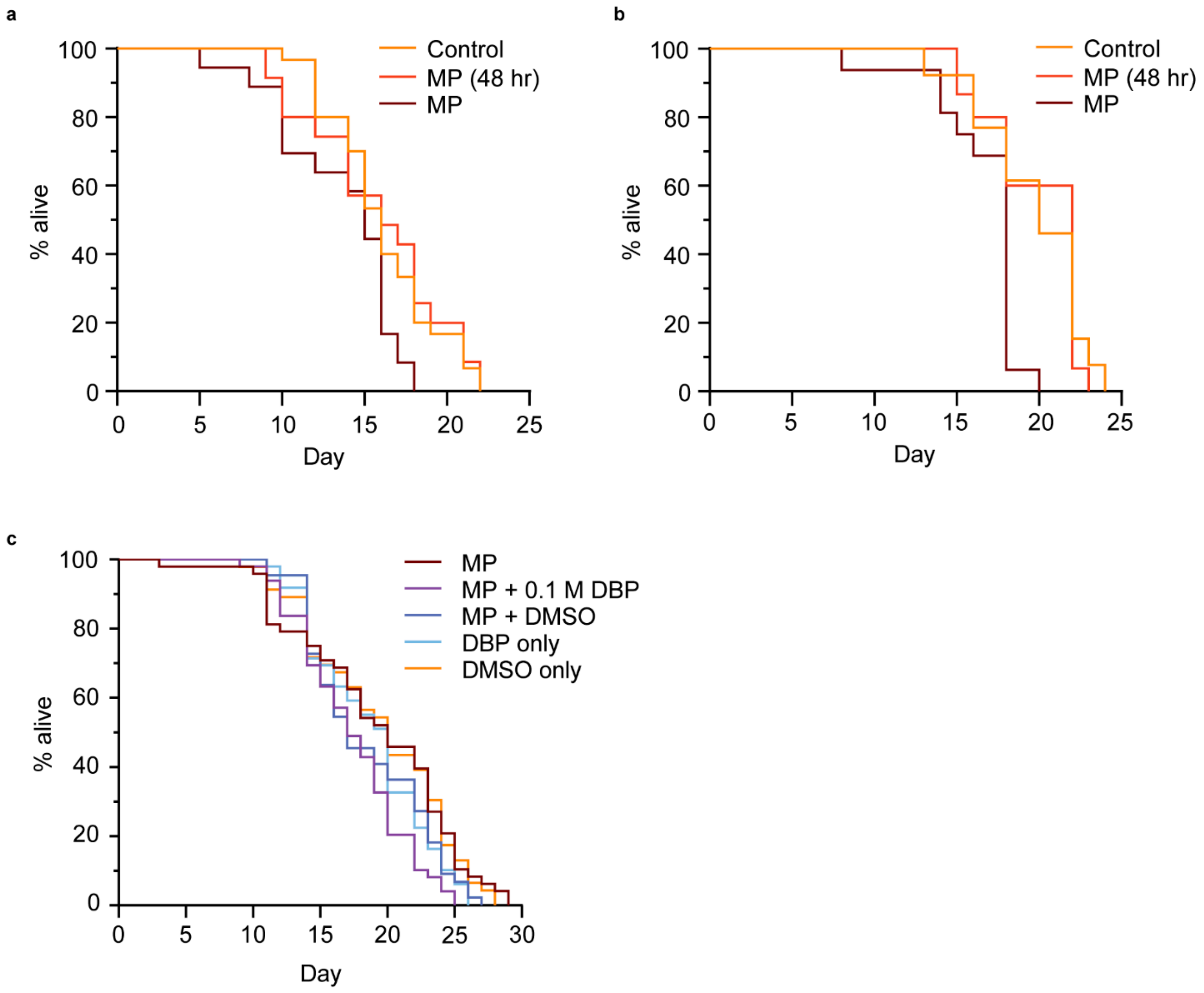

Supplemental Figure S6: Lifespan of *C. elegans* exposed to polystyrene (PS) microplastic (MP) and 0.1 M DBP. a) Survival assay for nematodes exposed to control (no MPs), 1 µg/L PS-MPs for 48 hours, or 1 µg/L PS-MPs continuously. n = 30, 35, 36. p = 0.0043. b) Same as a, n = 20, 19, 23. p = 0.0029. c) Survival assay for nematodes continuously exposed to 1 µg/L PS MPs, with and without being soaked in 0.1 M DBP or the solvent alone (100 % DMSO) for 24 hours. n = 48, 49, 44, 49, 46. p = 0.0136. Log-rank (Mantel-Cox).
